# A Molecular-Modeling Toolbox Aimed at Bridging the Gap between Medicinal Chemistry and Computational Sciences

**DOI:** 10.3390/ijms14010684

**Published:** 2013-01-04

**Authors:** Sameh Eid, Adam Zalewski, Martin Smieško, Beat Ernst, Angelo Vedani

**Affiliations:** Department of Pharmaceutical Sciences, University of Basel, Klingelbergstrasse 50, 4056 Basel, Switzerland; E-Mails: sameh.eid@unibas.ch (S.E.); adam.zalewski@unibas.ch (A.Z.); martin.smiesko@unibas.ch (M.S.); beat.ernst@unibas.ch (B.E.)

**Keywords:** computer-aided drug discovery, structure-based design, multi-dimensional QSAR, molecular dynamics, single-click molecular modeling

## Abstract

In the current era of high-throughput drug discovery and development, molecular modeling has become an indispensable tool for identifying, optimizing and prioritizing small-molecule drug candidates. The required background in computational chemistry and the knowledge of how to handle the complex underlying protocols, however, might keep medicinal chemists from routinely using *in silico* technologies. Our objective is to encourage those researchers to exploit existing modeling technologies more frequently through easy-to-use graphical user interfaces. In this account, we present two innovative tools (which we are prepared to share with academic institutions) facilitating computational tasks commonly utilized in drug discovery and development: (1) the *VirtualDesignLab* estimates the binding affinity of small molecules by simulating and quantifying their binding to the three-dimensional structure of a target protein; and (2) the *MD Client* launches molecular dynamics simulations aimed at exploring the time-dependent stability of ligand–protein complexes and provides residue-based interaction energies. This allows medicinal chemists to identify sites of potential improvement in their candidate molecule. As a case study, we present the application of our tools towards the design of novel antagonists for the FimH adhesin.

## 1. Introduction

Molecular modeling has become an integral part of drug discovery and development, with numerous documented examples of successful employment of computational approaches to answer key questions in the field of molecular design. For instance, structure-based design techniques, including small-molecule docking and scoring, can provide structural and energetic information on ligand–protein binding and, hence, guide the design of more potent candidate molecules [1,2]. Additionally, quantitative structure-activity relationships (QSAR) models can provide reliable estimates of binding affinities, particularly of hypothetical ligands—prior to their laborious and costly synthesis and biological testing [3,4]. Molecular dynamics (MD) simulations address more challenging questions regarding the dynamic nature of ligand–receptor interactions [5–11]. Overall, virtual screening can increase the efficiency and reduce time and cost of lead identification [12,13]. A number of commercially available software packages handle one or more of these tasks, e.g., the Schrödinger Suite [14], the Accelrys Discovery Studio [15], the SYBYL-X Suite [16] or the Molecular Operating Environment [17]. Furthermore, a wealth of modeling tools are available free-of-charge, including AutoDock for automated docking [18], Quasar^X^ for multi-dimensional QSAR [19], Desmond for molecular dynamics simulations [20] and DOCK Blaster for virtual screening [21].

Medicinal chemists involved in design of new ligands for some macromolecular target are nowadays knowledgeable of the binding site’s topology at the molecular level. This degree of familiarity with the target provides valuable guidance for modeling techniques, such as docking proposed ligands to that target or developing a QSAR for predicting binding affinities. Such optimized modeling methodologies could, in turn, guide the medicinal chemists’ decision making. However, making the best use of these and other modeling techniques requires a tedious and repetitive process of setting and calibrating parameters, as well as collecting and organizing the results. Designing an intuitive interface that encapsulates and hides the complexity of the underlying technologies from the end-user would, thus, motivate medicinal chemists to use modeling tools more frequently.

**Figure 1:**
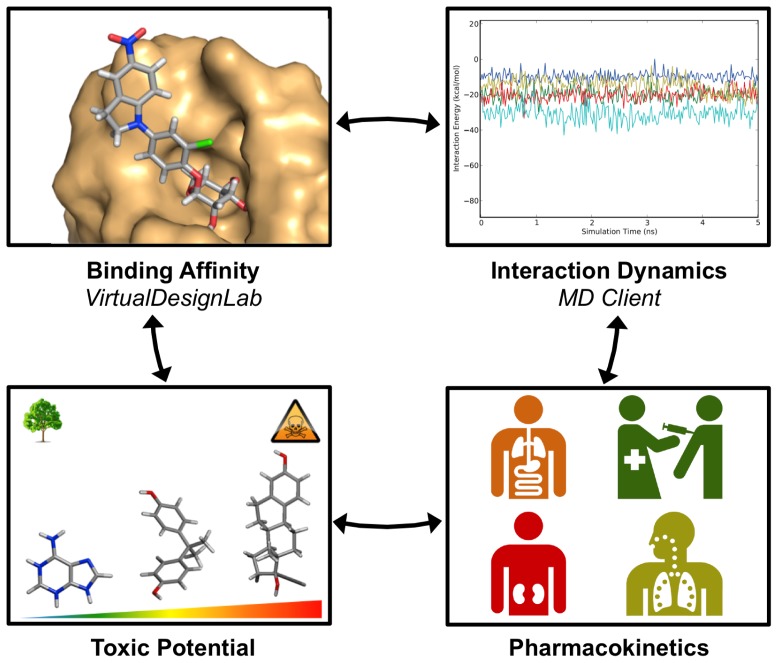
Tools presented in this article handle two common tasks in modern computer-aided drug design workflow. The *VirtualDesignLab* predicts binding mode and estimates the associated binding affinity of prospective ligands. The *MD Client* facilitates simulation and analysis of the dynamics in ligand–protein complexes. In concert with other software predicting pharmacokinetic (e.g., QikProp [25]) and toxicological profiles (e.g., the VirtualToxLab [26]), our tools equip medicinal chemists with a multi-purpose molecular-modeling kit.

In this article, we present two novel platforms addressing commonly required tasks in modern drug design workflow: the *VirtualDesignLab* for predicting binding mode and affinity and the *MD Client* for investigating interaction dynamics of ligand–protein complexes (Figure 1). We discuss the development of the underlying models and technologies used in both tools and demonstrate their recent employment in our lab for the design and optimization of novel antagonists for FimH [22–24], a bacterial lectin playing a crucial role in the initial stages of urinary tract infections. Since the goal of the present work is to develop versatile tools that can be easily tuned for any structure-based drug design project, we will conclude with reviewing the steps required to apply/extend our tools for use with other protein targets.

## 2. Methods

### 2.1. *VirtualDesignLab*

The *VirtualDesignLab* is an *in silico* tool developed at our institute (based on the *VirtualToxLab* framework [26] shared by the Biographics Laboratory 3R) simulating and quantifying the binding of small molecules to a macromolecular target. The technology employs automated, flexible docking combined with multi-dimensional quantitative structure-activity relationships (mQSAR). Controlled by an easy-to-use interface, the *VirtualDesignLab* allows medicinal chemists to perform quick and straightforward design, screening and structural inspection of any compound of interest [27].

In order to provide a reliable *in silico* affinity estimate for a given system, it is necessary to account for protein-ligand interactions, solvation and entropic phenomena. In our example system, FimH adhesin, we utilized a set of 108 compounds, along with their experimental affinity data, to develop and validate a corresponding mQSAR model (Table 1). When generating the model, the initial compound structures were constructed using the integrated model-building tool and then optimized with MacroModel [28]. Atomic partial charges were computed using the AMSOL package [29]. All structures were subjected to the conformational-searching algorithm ConfGen [30], resulting in sets of low-energy conformations for each molecule in aqueous solution. Energetically feasible binding conformations (within 10 kcal/mol from the lowest-energy structure) were identified by means of automated docking to two three-dimensional structures (“in” and “out” state, cf. below) of the FimH carbohydrate-binding domain. The employed alignment (Alignator) [31] and docking (Cheetah) [32] protocols allowed for flexibility of both ligand and the protein (induced fit), as well as dynamic solvation. Several templates (based on experimental structures) were used for the pre-alignment in order to account for distinct modes of binding to FimH (referred to as “in” and “out”) reported previously [23,33]. The underlying protein structures were retrieved from the Protein Data Bank (PDB codes 1UWF and 3MCY available at 1.69 Å and 2.90 Å resolution, respectively) and pre-processed (calculation of hydrogen-atom positions, hydrogen-bond network optimization, energy minimization) with the Protein Preparation Wizard in Maestro [34]. A total of 282 docking poses (allowing for multiple poses per ligand) comprising a 4D data set were then used as input (84 training and 24 test substances) for the mQSAR software Quasar [35] to generate a series of quasi-atomistic binding-site models. The underlying model families (comprising 200 members) were evaluated in consensus-scoring mode—along with a direct force-field scoring in Cheetah [32] and the comparison of a molecule’s interaction energy in a box of pre-equilibrated water and in the binding site. For validation, we additionally employed an alternative receptor-modeling concept, Raptor [28], featuring a substantially different scoring function.

**Figure 2:**
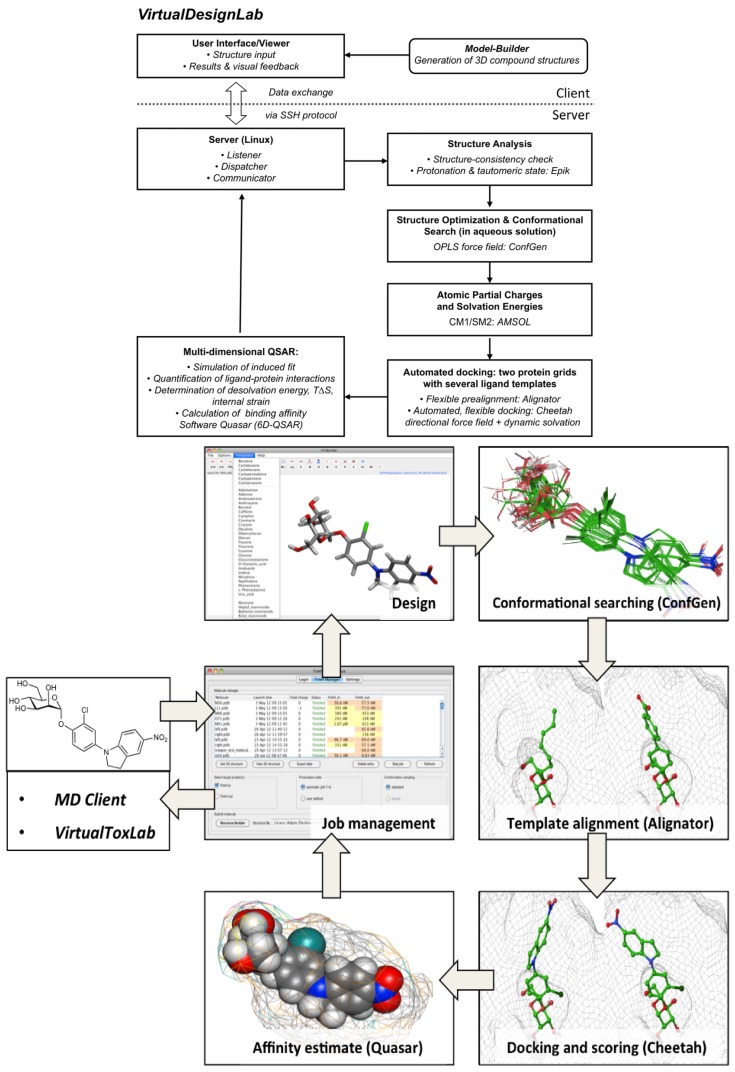
*VirtualDesignLab* flowchart (left) and an example of a typical workflow based on the FimH receptor (right). The compound of interest is designed using the built-in 3D model builder or imported from an external file. The main step involves the conformational sampling of the ligand in the protein’s binding pocket, where all feasible poses are retained and used as input for the subsequent mQSAR. Structure management options and job controls are all accessible from a central interface window. References to the individual pieces of software are given in text.

**Figure 3:**
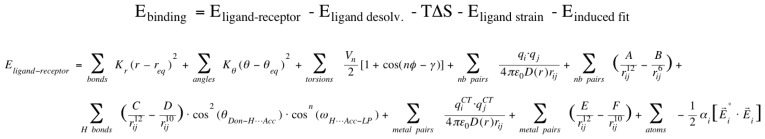
The equation for calculating the binding energy used in the *VirtualDesignLab/VirtualToxLab* and the directional force field employed in Cheetah and Quasar [32]. The individual terms—quantifying experimentally accessible quantities, such as bond lengths, bond angles, torsion angles, van der Waals contacts, geometries of hydrogen bonds, electrostatic and metal-ligand interactions, as well as ligand→protein polarization—are described in greater detail in the software documentation found at http://www.biograf.ch/index.php?id=software.

Every compound submitted to the *VirtualDesignLab* server (by means of imported PDB files or the integrated model builder) is subjected to identical protocols as those employed to train and validate the underlying mQSAR model(s) (Figure 2). The affinity is calculated based on multiple components of the binding energy (Figure 3). Protein–ligand interaction and internal strain energies (Cheetah and Quasar) are obtained using a directional force field with polarization terms [36]. The desolvation costs are calculated for the global minimum obtained from the conformational search, using a continuum solvation model. Loss of entropy is approximated from the number of rotatable bonds constrained upon binding to the protein. Induced-fit energy calculation is an inherent function of the Quasar algorithm. The affinity predictions are based on (up to) eight docking poses as obtained from Alignator/Cheetah (4D) and take into account (up to) six induced-fit mechanisms (5D) and two solvation (6D) scenarios in order to account for the unique properties of certain binding sites (e.g., the surface-exposed FimH binding pocket). Protein–ligand structures may be viewed (binding pocket) and/or downloaded (in PDB format) upon job completion. The latter files also serve as input for other software, including the *MD Client*.

### 2.2. *MD Client*

In addition to binding affinities estimated from mQSAR based on the docking simulations, medicinal chemists might wish to analyze the kinetic stability of ligand–protein interaction used by means of molecular-dynamics (MD) simulations. MD has been successful in studying structural fluctuations in proteins [37–39], lipids [40–42] and nucleic acids [43,44], as well as in the refinement of structures solved by X-ray crystallography and NMR [5]. Despite the availability of a wealth of software packages for performing MD simulations, (e.g., Desmond [20], Amber [45], CHARMM [46], GROMOS [47] and GROMACS [48]), the lengthy setup and laborious post-processing act as a barrier, preventing users from routinely utilizing these simulations. We therefore developed the *MD Client* to overcome this limitation by requiring as few settings as possible to quickly and reliably highlight basic features of the dynamics of the studied protein–ligand complex. Our *MD Client* is designed specifically for use by bench medicinal chemists interested in exploring ligand–protein interaction dynamics.

#### 2.2.1. The *MD Client* Interface

**Figure 4:**
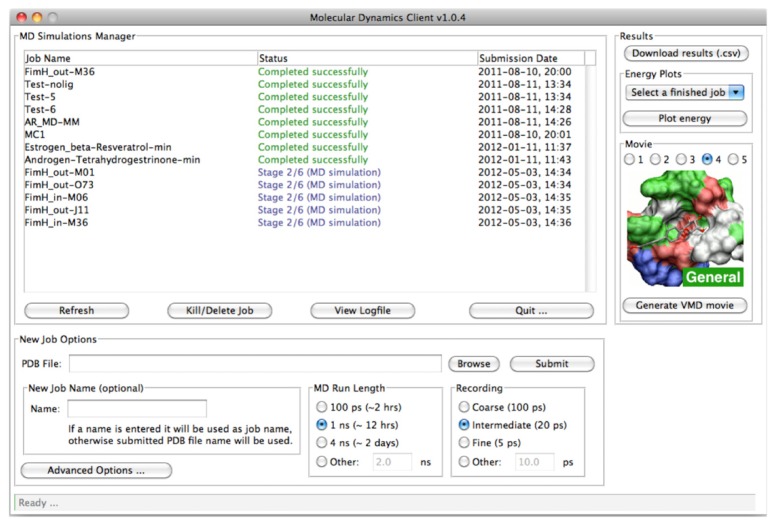
Appearance of the user interface of *MD Client*; top-left: list of jobs currently on remote server; bottom: basic simulation settings; and right: results download and analysis.

The *MD Client* utilizes a simple and intuitive GUI front-end and a more sophisticated back-end that handles all “under-the-hood” tasks, from cleaning the input structure to post-processing MD trajectory and gathering energy results. Both front- and back-end programs were developed in python 2.6 (http://www.python.org) using standard extensions, such as the *TkInter* GUI package and the *matplotlib* library for rendering interactive 2D plots. The front-end has been compiled for Mac OS X, Linux and Windows operating systems. The communication between the front-end and the back-end on the remote server is carried out via a Secure Shell (SSH) protocol. A molecular dynamics simulation of a ligand–protein complex (as obtained, for instance, from the *VirtualDesignLab*) is launched by a single-click in the *MD Client* interface. The *MD Client* provides control over the basic parameters of submitted MD simulation: namely its length and frequency of taking snapshots for subsequent energy analysis and movie production (Figure 4). The “Advanced Options”, button enables the user to control more details of the MD simulation; yet the default options are adequate in most cases. A list box keeps track of jobs currently on the server and their current status. The user can monitor the progress of running jobs or, when needed, terminate them at any stage.

MD simulations require different types of data input. The most important is the structure file containing input geometries of the ligand–protein complex. Currently, the *MD Client* accepts files with Protein Data Bank (PDB) format [49]. The *VirtualDesignLab* output structures (*i.e.*, ligand-protein complexes) can be directly used as input for the *MD Client*. When an MD simulation is completed, the user can download extracted frames as standard PDB files for viewing. For the 3D visualization of structures, several free 3D-rendering tools, such as Visual Molecular Dynamics (VMD) [50], are available. To facilitate the importing of MD trajectories into VMD, we added a functionality that automatically generates a VMD visualization-state file linked to the downloaded frames. The user can choose between different pre-defined visualization styles and simply click on “Generate VMD Movie” in the *MD Client* interface (Figure 4) to produce a file that can be loaded directly into VMD. This spares the user the time and effort needed to load individual PDB files and set up the view options in VMD. Most importantly, the user can use *MD Client’s* built-in plotting tool to analyze ligand–protein interaction dynamics (cf. Figure 7) and compare them amongst multiple systems, which we are going to demonstrate in the results section on selected antagonists binding to the FimH receptor.

#### 2.2.2. The *MD Client* Back-end

**Figure 5:**
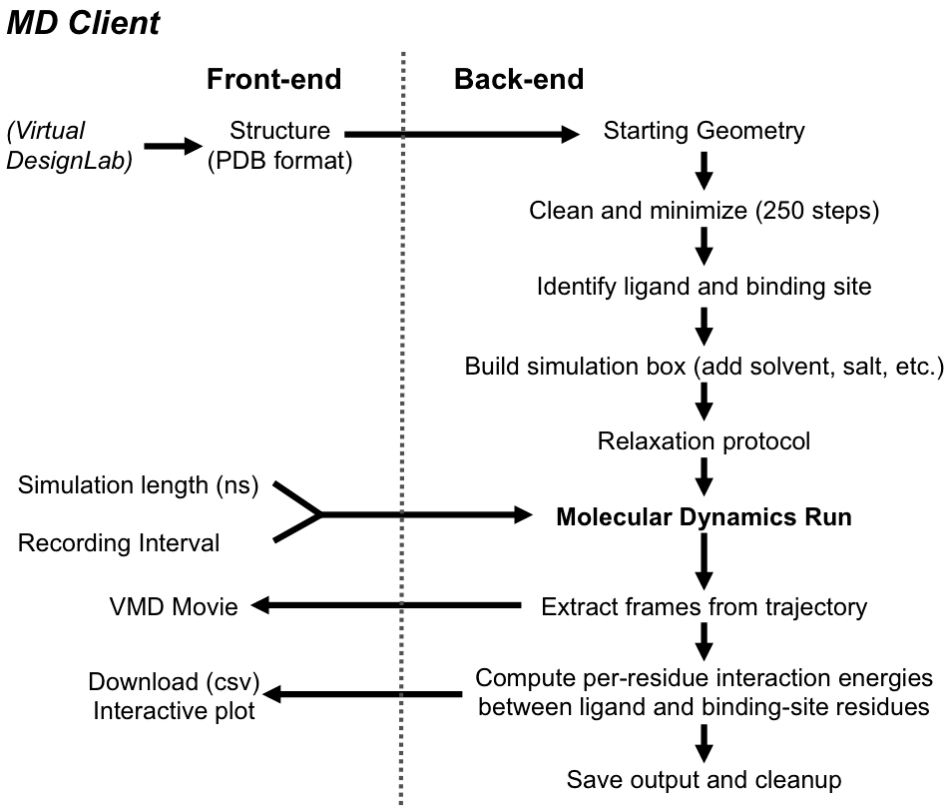
Workflow of the *MD Client* and communication between front-end and back-end.

The *MD Client* back-end resides on the remote server, where all computational jobs are to take place. It utilizes Schrödinger’s Python API (http://www.schrodinger.com/pythonapi) for reading structures, launching MD simulations and computing per-residue interaction energies. It receives input structure and primary simulation settings from the front-end. Figure 5 shows how the *MD Client* back-end processes input structures into useful quantities. It starts by constructing atom connectivity and bond orders for the submitted structure and doing a short energy minimization to relieve structural inconsistencies in bond lengths, angles, steric clashes, *etc.* The *MD Client* back-end automatically identifies the ligand-like molecule and defines binding site residues as all residues within 8 Å (default) from ligand atoms. This definition is employed for subsequent use in energy computations and movie production.

The *MD Client* back-end employs the Desmond package from the D. E. Shaw Research laboratory to perform the MD simulations [51,52]. Desmond and its source code are distributed under free license to non-commercial and academic users. It uses novel parallel algorithms and numerical techniques to achieve high performance and accuracy on platforms containing a large number of processors, but may also be executed on a single-processor computer [20]. Desmond’s System Builder soaks the submitted ligand–protein complex into a TIP3P water box extending 10 Å beyond any of the complex’s atoms. It adds counter ions to neutralize the simulation box and 0.15 M sodium and chloride ions to approximate physiological conditions. The complex is first minimized to a convergence gradient threshold of 1.0 kcal/(mol·Å). The molecular-dynamics protocol utilizes the OPLS2005 force field and the NPT ensemble (constant number of particles, pressure and temperature) at 300 K, with periodic boundary conditions. The production run of the user-defined length is preceded by 24 ps of the Desmond default relaxation protocol. After completion of the MD simulation, the *MD Client* back-end extracts frames at the user-defined intervals and saves them as standard PDB files. The user can download these frames for later viewing and analysis. Finally, the extracted frames are analyzed using the component-interactions script in Maestro [34] to compute interaction energies between the ligand and individual amino acids defining the binding site along the MD simulations. Ligand-residue interaction energies are calculated as the sum of the (OPLS2005) van der Waals and electrostatic terms. This dynamic-interaction profile is saved as a time series in a comma-separated-values (csv) file for subsequent download by the user or for interactive plotting and analysis using the *MD Client* front-end interface.

## 3. Results and Discussion

### 3.1. *VirtualDesignLab*

**Figure 6:**
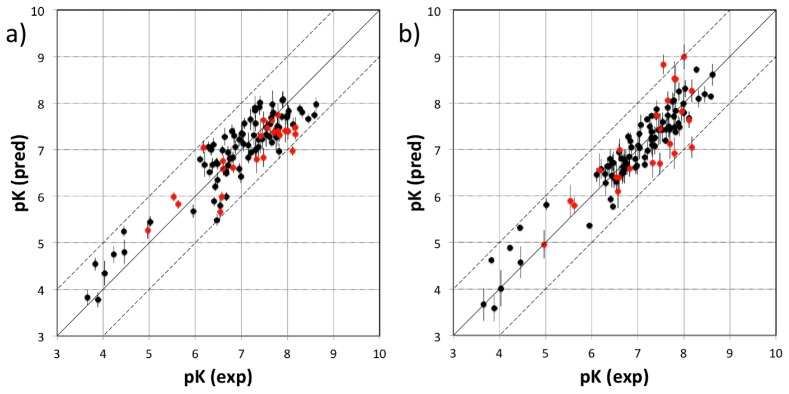
Comparison between experimental (horizontal axis) and predicted pK values (vertical axis) for (**a**) the Quasar model and (**b**) the Raptor model. Black and red points represent compounds of the training and test set, respectively. Vertical bars indicate the estimated standard deviations of the prediction. Dashed lines are drawn at factors of 10 from the experimental value.

The current FimH Quasar model for the *VirtualDesignLab* was established based on structural and biological data of 108 mannose-based inhibitors (with IC_50_ values ranging from 220 μM to 2.4 nM) displaying diverse PK/PD profiles. Compound synthesis, biological assays [53] and model development were performed in-house, ensuring consistency of all results. Table 1 shows the structures, experimental and predicted affinities of compounds used for developing the model. The QSAR model based on a genetic algorithm converged at a cross-validated *r*^2^ of 0.805 and yielded a predictive *r^2^* of 0.596 (Figure 6a). The only modest value of the predictive *r^2^* is a consequence of the relatively narrow range of test compound affinities (as some substances were necessary for the training set due to their structural uniqueness). The performance of the model is therefore better reflected by the individual predictions (23 out of 24 test substances within a factor of 10 from their experimental affinity). We further challenged the FimH model by using Y-scrambling and consensus scoring with the software Raptor (dual-shell 5D-QSAR; Figures 6b and S1) [54]. All tests, including the processing of additional external compounds, confirmed the predictive power of the mQSAR model-based framework.

The *VirtualDesignLab* is aimed at predicting the binding affinity for a given compound within a factor of 10 from the experimental value. Currently, the affinity prediction for a single compound requires approximately one hour of CPU time—a good balance between accuracy and processing time. Special treatment may, however, be required for compounds retaining flexibility upon binding. In such cases, improved entropy estimation (a method is currently in development at our institute) or a non-static approach, such as the one offered by the *MD Client* (cf. below), may be necessary. We would like to emphasize that the framework is independent of the FimH mQSAR model (presented here), as it only requires the generation and validation of a new QSAR model for any target protein of interest. This can be developed using by freely available software, e.g., Quasar [35].

### 3.2. *MD Client*

**Figure 7:**
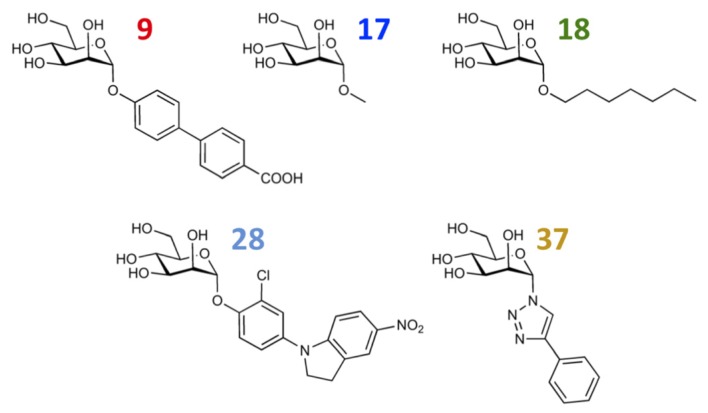

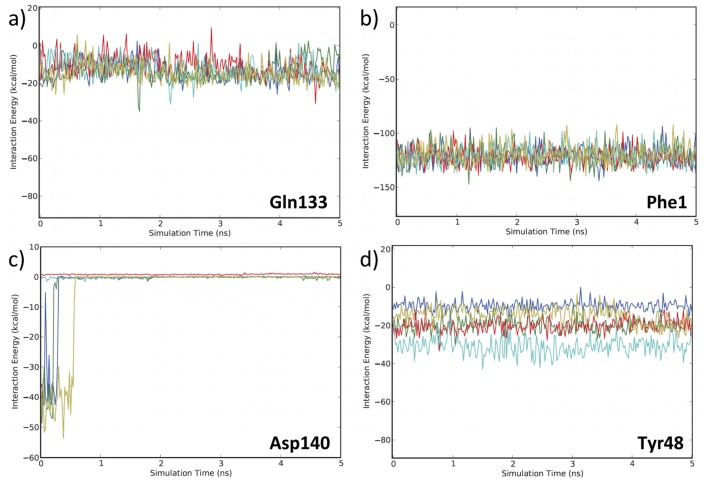
Dynamic per-residue interaction plots for five FimH ligands generated by the interactive plotting feature of *MD Client*; (**a**) Gln133, (**b**) Phe1, (**c**) Asp140 and (**d**) Tyr48. Vertical axis: protein–ligand interaction energies (kcal/mol); horizontal axis: molecular dynamics simulation time (ns). The colors mark the individual compounds shown above.

In the *MD Client*, the outcome of an MD simulation includes a set of frames extracted from the MD trajectory and a dynamic-interaction profile comprising per-residue interaction energies between ligand and protein for all time points. An interactive plot of computed interaction profiles is readily accessible from the *MD Client* interface (Figure 7). The plot created by the *matplotlib* python extension is cumulative, *i.e.*, it can incorporate dynamic profiles from several simulations in the same plot with automated coloring and legend generation. Comparing dynamic profiles of different simulations may provide valuable clues, for instance, about interaction modes of different ligands and/or key residues in ligand recognition and binding. We chose five structurally distinct FimH ligands (9, 17, 18, 28 and 37) to demonstrate the usefulness of dynamic-interaction profiles. Examination of their dynamic interaction energies with two key FimH residues (Gln133 and Phe1) indicates that these interactions are maintained throughout the entire simulation and that they don’t significantly differ among different ligand classes (Figure 7a,b). These residues are typically involved in an extended hydrogen-bond network with the mannose moiety common to FimH binders. The profiles also show that the interaction with the N-terminal NH_3_^+^ moiety of Phe1 results in a considerably larger contribution to the binding enthalpy compared to Gln133. Automated docking of FimH ligands typically predicts a hydrogen bond from the 3-OH of the mannose moiety to the Asp140 residue to be thermodynamically favorable. Interestingly, this hydrogen bond does not seem to be kinetically stable, since it is broken within the first 0.5 ns and is never re-established throughout the entire simulation as can be observed in the profiles of all studied compounds (Figure 7c).

**Figure 8:**
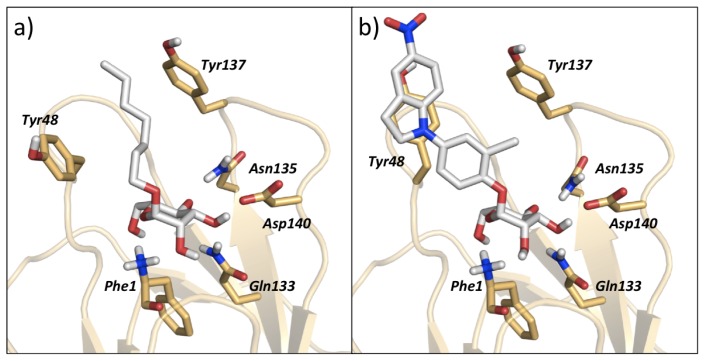
Illustration of (**a**) *in* binding mode of 18 and (**b**) *out* binding mode of 28, within the tyrosine gate of FimH binding site. References to different binding modes of FimH ligands are given in the methods section.

The various classes of FimH ligands differ in their interaction patterns with the so-called *tyrosine gate* lined by Tyr48 and Tyr137 located at the entrance to the mannose-binding site (Figure 8) [23]. The interaction dynamics show that compound 28 exhibits the strongest interaction with Tyr48, which could be explained by its unique scaffold that allows a preferred interaction with the tyrosine side chain (Figure 7d). Compounds 9 and 18 display a favorable interaction with Tyr48, yet of lower magnitude than 28, which explains the superior affinity of the latter (see Table 1). Finally, two ligands seem to lack this favorable interaction with Tyr48; namely 17 and 37, which also coincides with their relatively lower affinities. This could be rationalized by the lack of a side chain capable of interacting with the Tyr48 in 17 and the inherent rigidity of 37 due to a lack of glycosidic oxygen in the linker between mannose and the aromatic aglycone.

## 4. Software Extension/Repurposing

The philosophy behind all our software is to allow for extendibility, as well as redirection, towards different targets of interest. In the following, we provide a brief overview of the corresponding requirements.

### 4.1. *VirtualDesignLab*

Assuming that a decent number of ligands with experimental affinity data are available for the given target, a three-dimensional protein-ligand structure that will serve for automated, flexible docking is required. These are usually obtained by means of crystallography or homology modeling and must typically be further refined (addition of hydrogen atoms, completion of missing amino-acid residues or their side chains, completion/generation of the solvent shell, energy minimization). These tasks can be accomplished through numerous, freely accessible computational tools. With the structure at hand, potential binding poses of all tested compounds need to be obtained. For this step, any flexible-docking software may be employed, including Alignator/Cheetah discussed in this article. The ensemble of potential binding modes can be compiled into a 4D data set to serve as input for the generation of the binding site surrogate. Though this task is best handled using the Quasar software, a QSAR model of different origin could also potentially be utilized. It should be noted, however, that even though structure preparation and pose generation are relatively simple tasks requiring no more than a few days of work, developing and validating a robust and reliable QSAR model is a complex and lengthy procedure. Also, given the vast diversity of the computational methods, personal communication with the authors of this article would likely be necessary in order to integrate a QSAR model with the *VirtualDesignLab* framework.

### 4.2. *MD Client*

The *MD Client* relies on the Desmond package at its back-end terminal to perform MD simulations. Desmond can be obtained free of charge for academics and non-commercial users. Once a working Desmond installation is available, the user needs to point the *MD Client* to where the back-end is located by providing the necessary SSH credentials. The *MD Client* can basically take it from there, since it has internal routines for identifying protein and ligand, job submission and monitoring, as well as calculating, organizing and plotting the interaction energy results.

## 5. Conclusions

Over the past three decades, much progress has been made in developing and validating innovative computational algorithms for common drug design-related tasks. In their perspective on the future of medicinal chemistry, Satyanarayanajois and Hill [55] stated that emerging medicinal chemists should additionally acquire “computational and cheminformatics acumen considerably greater than in years past”. In a related analysis, Ritchie and McLay [56] concluded that the goal of encouraging medicinal chemists to rely more on computational chemistry tools could be best achieved via specially designed tools that are “well-thought-out, suitable for their needs, able to generate useful, timely and valid results”. Similarly, we trust that adopting this strategy will ultimately maximize the benefit of state-of-the-art modeling technologies in the field of drug design and development.

To this end, we designed versatile single-click tools to assist medicinal chemists in performing two routine modeling tasks: the *VirtualDesignLab* for predicting binding mode and affinity of potential drug candidates and the *MD Client* for investigating dynamic behavior and energetics of ligand-protein complexes. Thanks to their modular design based mainly on self-developed algorithms, our tools allow easy modification, extension, as well as reorientation towards other targets and platforms of interest. Our group previously introduced the *OpenVirtualToxLab* for prediction of the toxic potential of drug candidates and made it freely available to academic organizations [57]. The two new tools introduced in this article, *VirtualDesignLab* and *MD Client*, can also be made available on request. Our future plans for *MD Client* include adding support for more molecular dynamics packages, as well as more analysis functionalities (for instance, surface area and entropy computations) to give more insight into ligand–protein interaction processes.

In closing, we wish to emphasize that our tools are not intended to, neither can they, replace the expert molecular modeler. In fact, their main purpose is to facilitate handling routine drug-design related tasks, thus leaving the more time-consuming detailed investigation only for interesting cases. Our vision is to place our interfacing technologies right on the medicinal chemists’ workbench and keep all the complicated ‘machinery’ on a transparently maintained server. However, the simplicity of such tools, although tempting, should never cloud the medicinal chemists’ judgment. On the contrary, medicinal chemists should always employ their expertise to question the results obtained from such tools.

## Supplementary Information



## Figures and Tables

**Table 1:** Structures and binding affinities (pIC50: negative logarithm of IC_50_ [M]) for 52 compounds employed to develop the QSAR model. The remaining data cannot be disclosed at this time, due to pending patent applications.

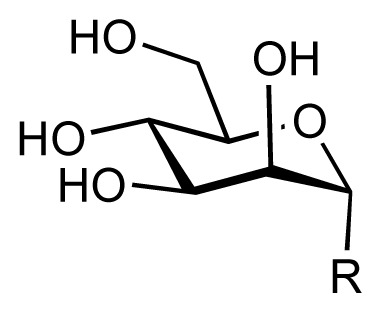									

	R	Exp. affinity	Pred. affinity	Residual		R	Exp. affinity	Pred. affinity	Residual
**1**	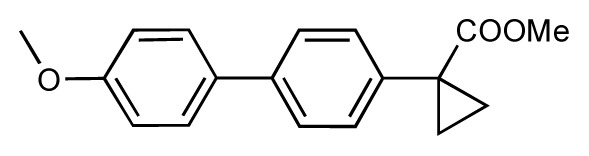	7.3	7.6	0.3	**2**	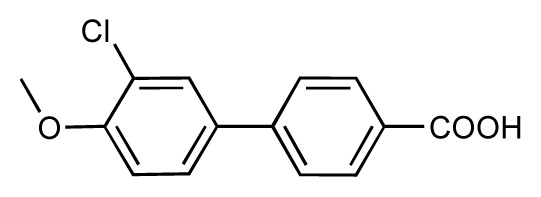	7.0	7.4	0.4
**3**	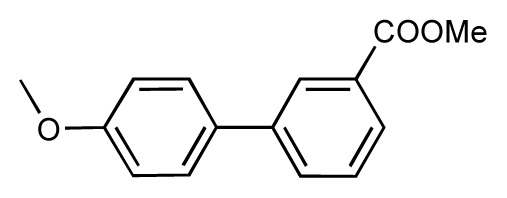	7.5	7.5	0	**4**	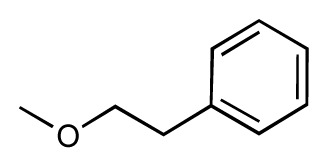	6.7	6.5	−0.2
**5**	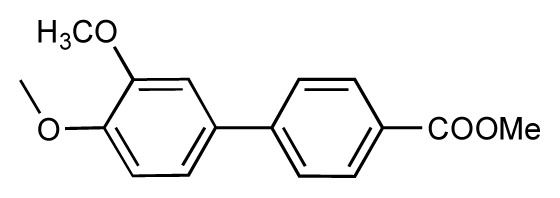	8.5	7.7	−0.8	**6**	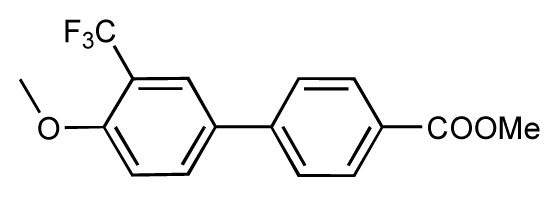	8.6	7.8	−0.8
**7**	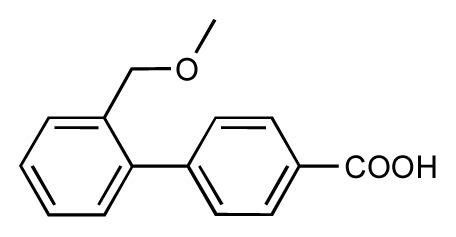	6.2	6.7	0.5	**8**	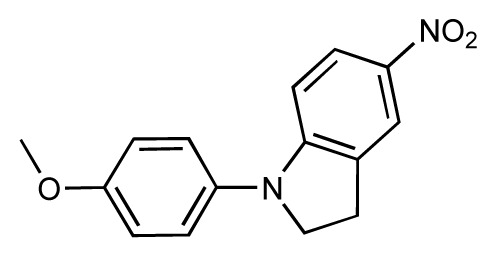	7.8	7.0	−0.8
**9**	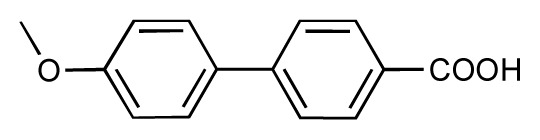	8.0	7.4	−0.6	**10**	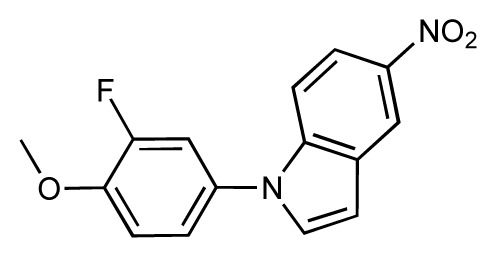	7.5	7.2	−0.3
**11**	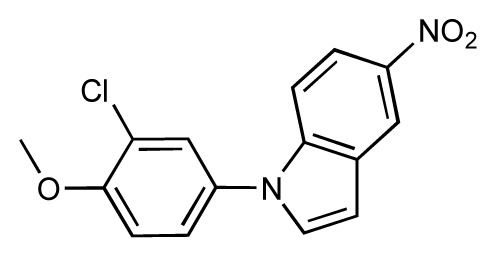	7.8	7.3	−0.5	**12**	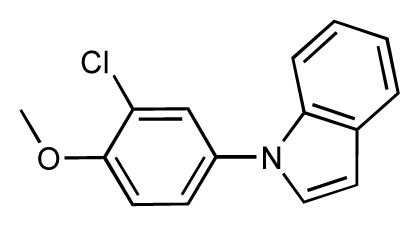	8.1	7.0	−1.1
**13**	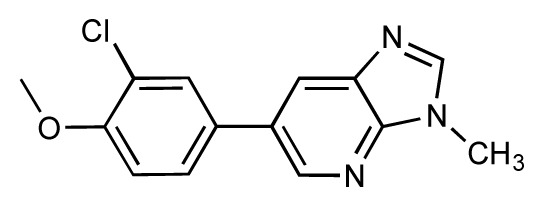	7.8	7.5	−0.3	**14**	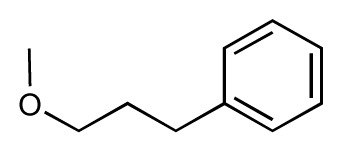	6.6	7.0	0.4
**15**	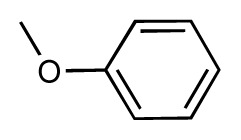	6.8	6.6	−0.2	**16**	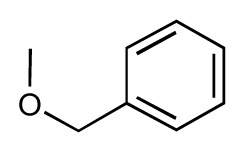	6.4	6.2	−0.2
**17**	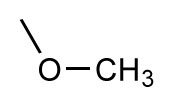	5.5	6.0	0.5	**18**	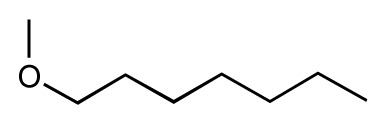	7.2	6.9	−0.3
**19**	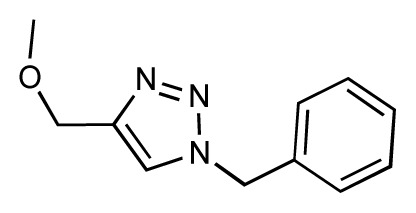	6.2	7.1	0.9	**20**	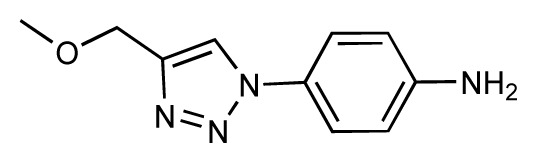	6.4	6.7	0.3
**21**	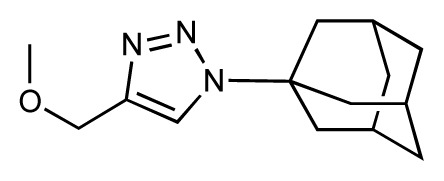	6.9	7.1	0.2	**22**	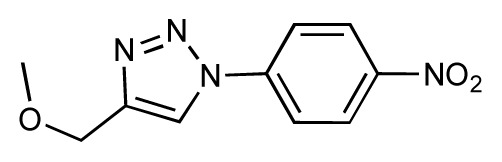	6.4	7.1	0.7
**23**	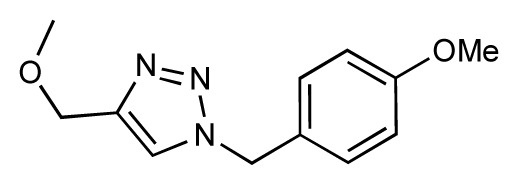	6.3	7.1	0.8	**24**	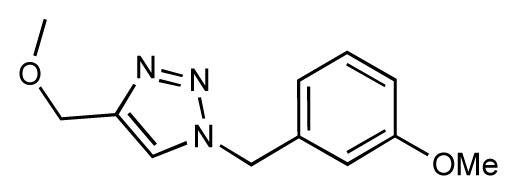	6.3	7.0	0.7
**25**	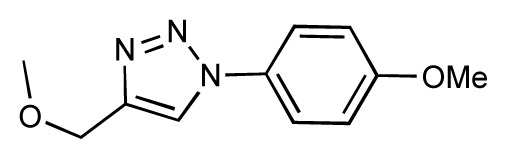	6.8	7.4	0.6	**26**	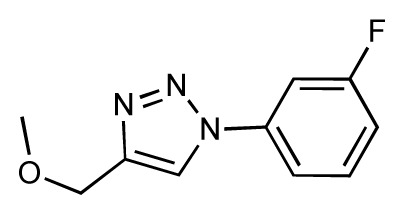	6.5	6.7	0.2
**27**	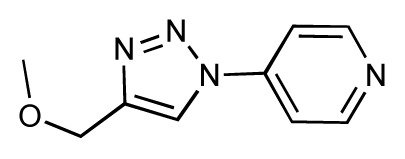	7.2	6.8	−0.4	**28**	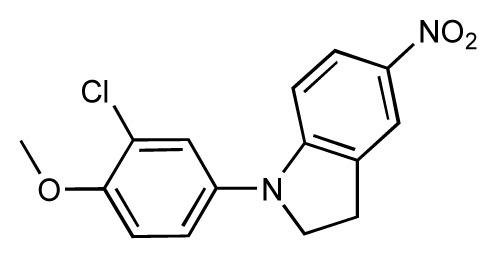	8.6	8.0	−0.6
**29**	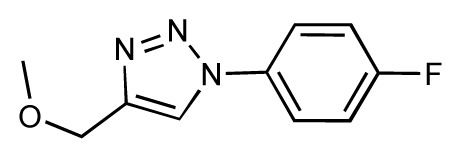	6.1	6.8	0.7	**30**	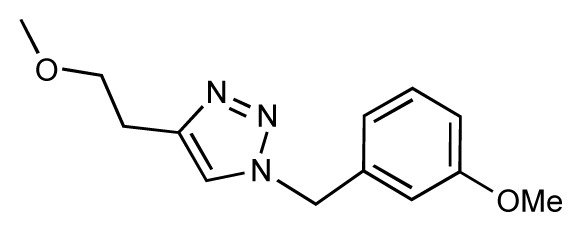	6.6	7.3	0.7
**31**	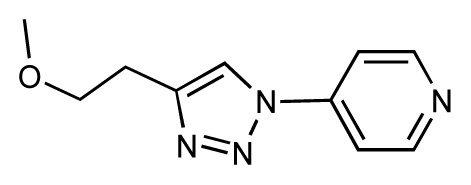	6.8	6.8	0	**32**	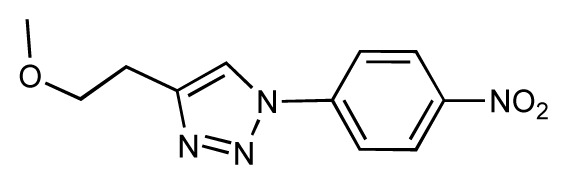	7.0	7.2	0.2
**33**	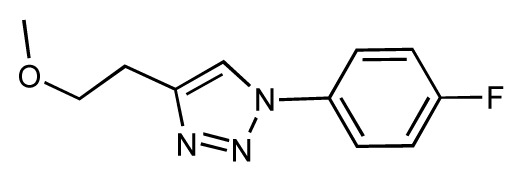	6.7	6.9	0.2	**34**	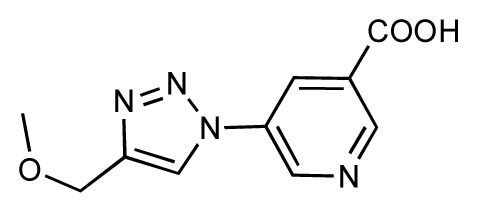	6.8	6.8	0
**35**	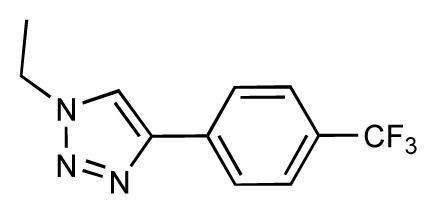	6.7	6.7	0	**36**	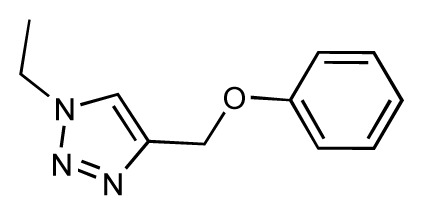	6.5	6.3	−0.2
**37**	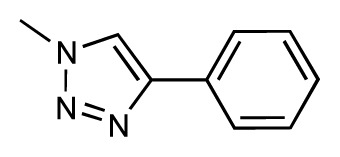	6.6	6.8	0.2	**38**	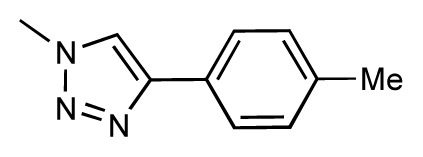	6.6	6.6	0
**39**	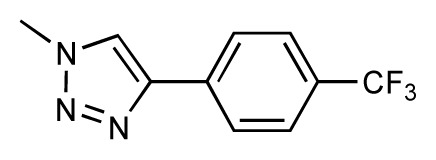	6.8	7.3	0.5	**40**	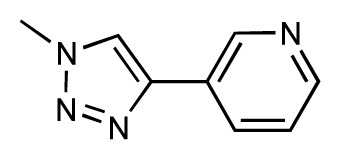	6.7	6.5	−0.2
**41**	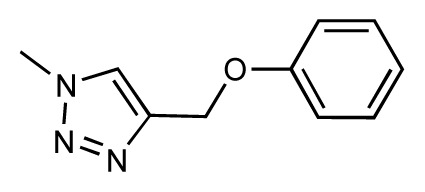	6.3	6.5	0.2	**42**	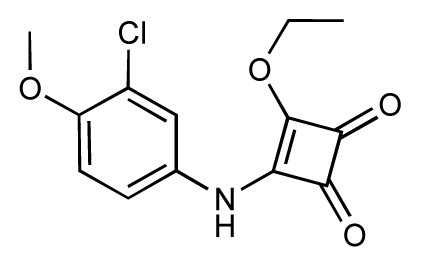	8.0	7.7	−0.3
**43**	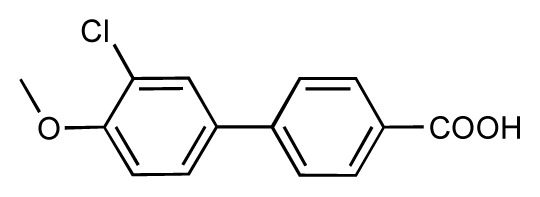	8.2	7.5	−0.7	**44**	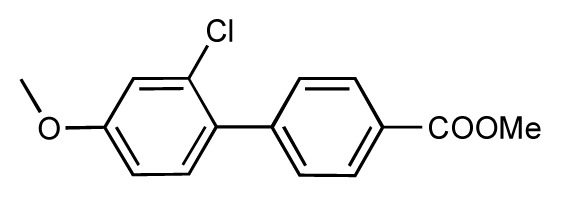	7.7	7.6	−0.1
**45**	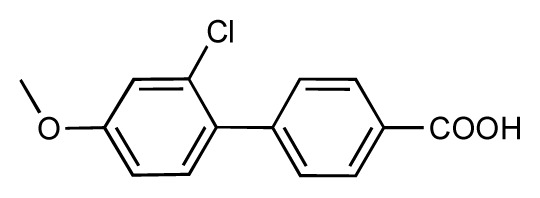	7.6	7.5	−0.1	**46**	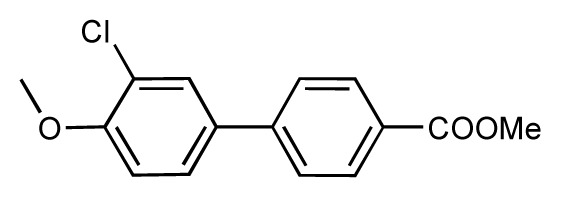	8.3	7.8	−0.5
**47**	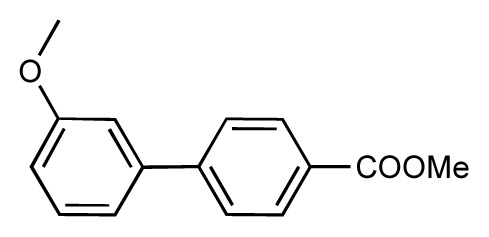	7.7	7.4	−0.3	**48**	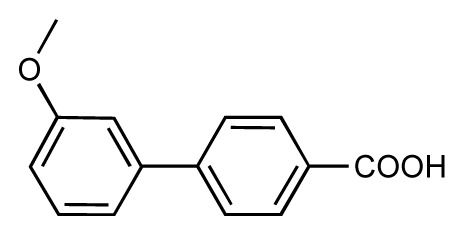	7.4	7.3	−0.1
**49**	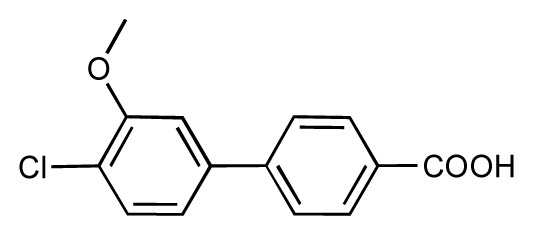	7.5	7.3	−0.2	**50**	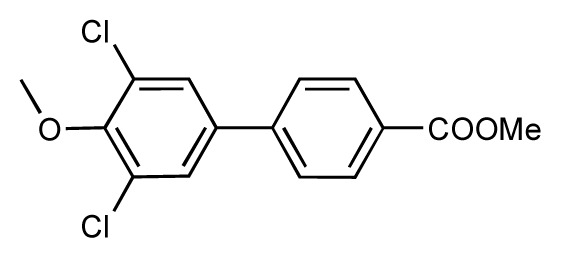	7.8	7.4	−0.4
**51**	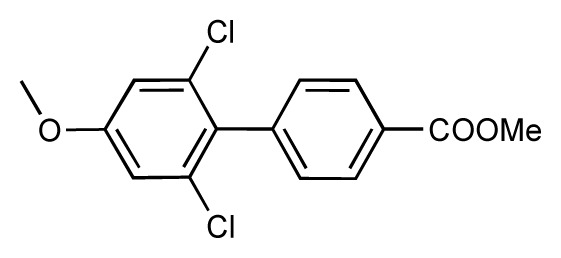	8.3	7.9	−0.4	**52**	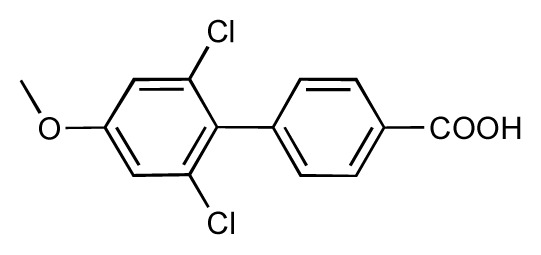	8.0	7.4	−0.6
