# Supplementary material for: A Molecular-Modeling Toolbox Aimed at Bridging the Gap between Medicinal Chemistry and Computational Sciences
Source: Int J Mol Sci. 2013 Jan 4;14(1):684–700. doi: 10.3390/ijms14010684 (PMC3565289; doi:10.3390/ijms14010684)
Supplement: Supplementary file 1 [file ijms-14-00684-s001.pdf]

Supplementary Information

Figure S1. Comparison between experimental (horizontal axis) and predicted (vertical axis) pK values for (**a**) and (**b**) scramble tests, and (**c**) additional set of test compounds. Points represent training (black) and test (red) compounds. Cross-validation and prediction coefficients are given as q2 and p2, respectively.

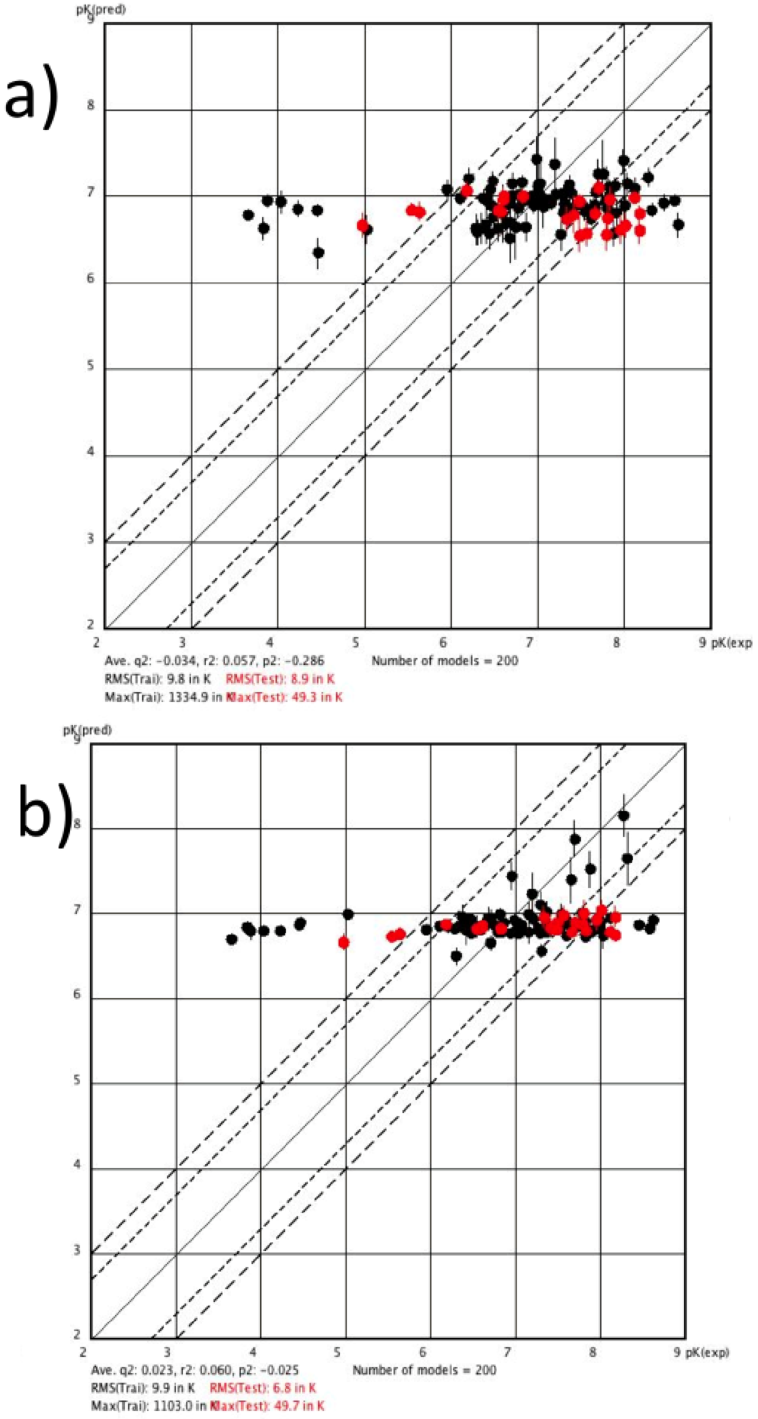

Figure S1. *Cont.*

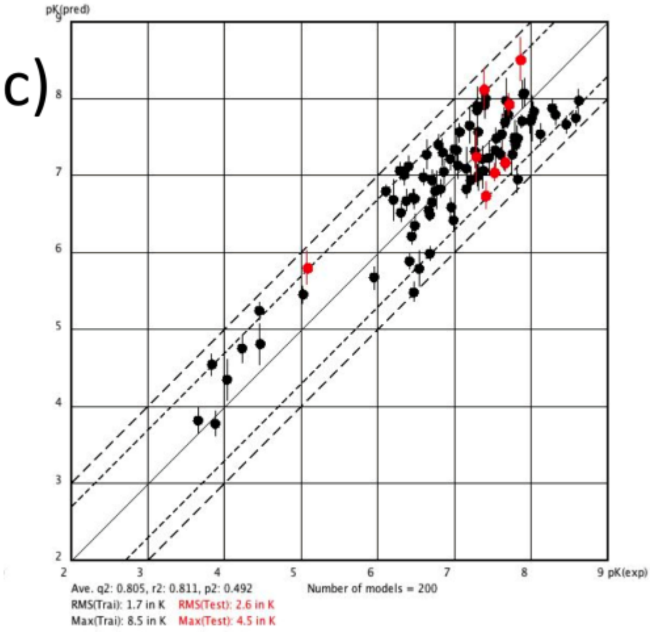

© 2013 by the authors; licensee MDPI, Basel, Switzerland. This article is an open access article distributed under the terms and conditions of the Creative Commons Attribution license (http://creativecommons.org/licenses/by/3.0/).
